# First Report of the Presence of Hepatitis E Virus in Scottish-Harvested Shellfish Purchased at Retail Level

**DOI:** 10.1007/s12560-018-9337-5

**Published:** 2018-02-13

**Authors:** Zoe O’Hara, Claire Crossan, John Craft, Linda Scobie

**Affiliations:** School of Health and Life Sciences, Glasgow Caledonian University, Glasgow, Scotland, UK

**Keywords:** Hepatitis E virus, HEV, Genotype 3, Bivalve mollusks, Shellfish, Contamination

## Abstract

Shellfish samples (*n* = 310) purchased from local supermarkets were analysed for the presence of hepatitis E virus (HEV) by nested RT-PCR and real-time qRT-PCR. Overall, 2.9% of samples tested positive for the presence of HEV. Phylogenetic analysis of HEV sequences revealed all as being genotype 3 HEV. This is the first report of the detection of HEV in commercially sold shellfish in Scotland. These findings may encourage further research that will help address the gaps in the knowledge in respect to foodborne transmission of HEV in Scotland and the rest of the United Kingdom.

## Introduction

The Scottish aquaculture industry produces approximately 7700 tonnes of mussels annually with the aim to increase this to 13,000 tonnes by 2020 (http://www.gov.scot/Topics/marine/Fish-Shellfish), and is worth approximately £11.7 million at first sale value (Munro & Wallace 2016). In the United Kingdom, a steady increase in the number of cases of non-travel-related hepatitis E virus (HEV) infection has been observed in recent years (Grierson et al. 2015; Crossan et al. 2015). A substantial increase in laboratory reports of non-travel-associated HEV infections in Scotland has been observed with the number of cases of HEV infection increasing from 13 in 2011 to 206 in 2016 (http://www.hps.scot.nhs.uk/ewr/article.aspx). In Scotland, routes of transmission are not fully understood but a number of potential risk factors have been identified. Foodborne transmission of HEV is a major route of infection in Europe with contaminated food products such as leafy green vegetables, sausages and soft fruits being potential sources of transmission (Donnelly et al. 2017). Demonstrated by a multivariate study, the consumption of processed pork products was found to be significantly associated with indigenous genotype 3 HEV infection (Said et al. 2014). Consumption of shellfish has also been identified as a possible risk factor of foodborne transmission of HEV to humans (Said et al. 2009). There are a number of studies from European countries documenting the presence of enteric viruses, including HEV, in both wild and commercially grown mussels (Chironna et al. 2002; Myrmel et al. 2004; Diez-Valcarce et al. 2012; Krog et al. 2014; Suffredini et al. 2014; Mesquita et al. 2016; La Rosa et al. 2017). To date, only one Scottish study has reported the presence of HEV in wild Scottish mussels; however, these were grown in unregulated waters and were not destined for retail (Crossan et al. 2012). No studies in Scotland have been undertaken to assess the presence of HEV in commercially harvested *Mytilus edulis* and *Crassostrea gigas* at point of sale in retailers. Despite the increase in the number of cases of autochthonous hepatitis E infection in Scotland, there is a distinct lack of information regarding transmission routes. In the present study, Scottish-harvested *M. edulis* and *C. gigas* were purchased from local supermarkets and analysed for the presence of HEV RNA.

## Materials and Methods

### Origin and Processing of Shellfish Samples

310 live shellfish samples (270 blue mussels and 40 Pacific oysters) were purchased from 4 local supermarkets and a local fishmonger and processed in accordance to ISO/TS15216-1 (ISO 2013). Shellfish were purchased at different time periods. Viral RNA was extracted from shellfish supernatants using QIamp Viral RNA Mini Kit (Qiagen, Hilden, Germany). Eluted viral RNA was used immediately or stored at − 80 °C until further use.

### Detection and Amplification of HEV RNA by Nested RT-PCR and Real-Time qRT-PCR

Shellfish samples were assayed for HEV RNA using a previously described nested RT-PCR with universal oligonucleotides targeting the ORF2 region of the HEV genome (Table 1) (Erker et al. 1999). The World Health Organization (WHO) hepatitis E virus 1st International genotype 3a standard (PEI code 6329/10) has been assigned a unitage of 250,000 International Units per millilitre (IU/ml) and was utilised as the positive control for all RT-PCR reactions. The limit of detection for the assay is 2.5 IU/ml.

**Table 1:** Oligonucleotide sequences used for the detection and amplification of HEV in mussel and oyster tissues

Target region	5′–3′ primer sequences	Length (bp)	Cycle	Primer position^a^	Reference
ORF2	con-s1: GACAGAATTRATTTCGTCGGCTGG	192	First	6341-6364	Erker et al. (1999)
	con-a1: CTTGTTCRTGYTGGTTRTCATAATC			6513-6537	
	con-s2: GTYGTCTCRGCCAATGGCGAGC	145	Nested	6390-6411	
	con-a2: GTTCRTGYTGGTTRTCATAATCCTG			6510-6534	

^a^Primer positions are relative to HEV Burma strain M73218

All samples positive for HEV RNA by nested RT-PCR were assayed for HEV RNA by real-time qRT-PCR with the hepatitisE@ceeramTools HEV detection kit (BioMérieux, France). The World Health Organization (WHO) hepatitis E virus 1st International genotype 3a standard (PEI code 6329/10) was used to generate a standard curve for HEV RNA quantitation. The limit of detection of the hepatitis@ceeramTools assay is 25 IU/ml (Mokhtari et al. 2013).

### Sequencing and Phylogenetic Analysis

All samples positive for the presence of 145-bp PCR fragments were subject to Sanger sequencing (GATC, Cologne, Germany). Sequences were augmented with additional ORF2 sequences derived from wild mussels (*n* = 12) (Crossan et al. 2012). Proposed reference sequences for HEV genotypes and HEV subtypes were included in the analysis (Lu et al. 2006; Smith et al. 2016). Genotype 3 sequences from Scottish swine and human patients were incorporated into the analysis (Crossan et al. 2014). Nucleotide sequences were aligned using EMBL-EBI CLUSTAL omega (https://www.ebi.ac.uk/Tools/msa/clustalo/) and the alignment transformed into PAUP/NEXUS format with EMBL-EBI EMBOSS SEQRET (https://www.ebi.ac.uk/Tools/sfc/emboss_seqret/). The sequences were used for Bayesian inference analysis with MrBayes v3.2.1 using the Markov chain Monte Carlo (MCMC) method (Ronquist & Huelsenbeck 2003). The majority rule consensus tree was rendered with FigTree v1.4.2 (http://tree.bio.ed.ac.uk/software/figtree/).

## Results

Overall, 9 out of 310 (2.9%) of shellfish samples were positive for HEV by nested RT-PCR. The presence of HEV was detected in 8 of the 270 (2.9%) *M. edulis* samples and 1 of the 40 (2.5%) Pacific oysters by nested RT-PCR.

Of the 9 shellfish samples positive for HEV by nested RT-PCR, 8 had material available for assay by qRT-PCR. Two samples were positive for HEV RNA with the hepatitisE@ceeramTools HEV detection assay. However, the virus titre could be quantitated for only 1 sample and was calculated at 62.4 IU/ml.

HEV sequences were successfully obtained from 3 out of the 9 samples that were positive for HEV by nested RT-PCR. The 3 sequences were derived from mussels, all of which were purchased from three different retailers but initially processed and dispatched from a central processing site. The inability to successfully clone and sequence samples with low virus levels has been described previously (Grierson et al. 2015).

**Fig. 1:**
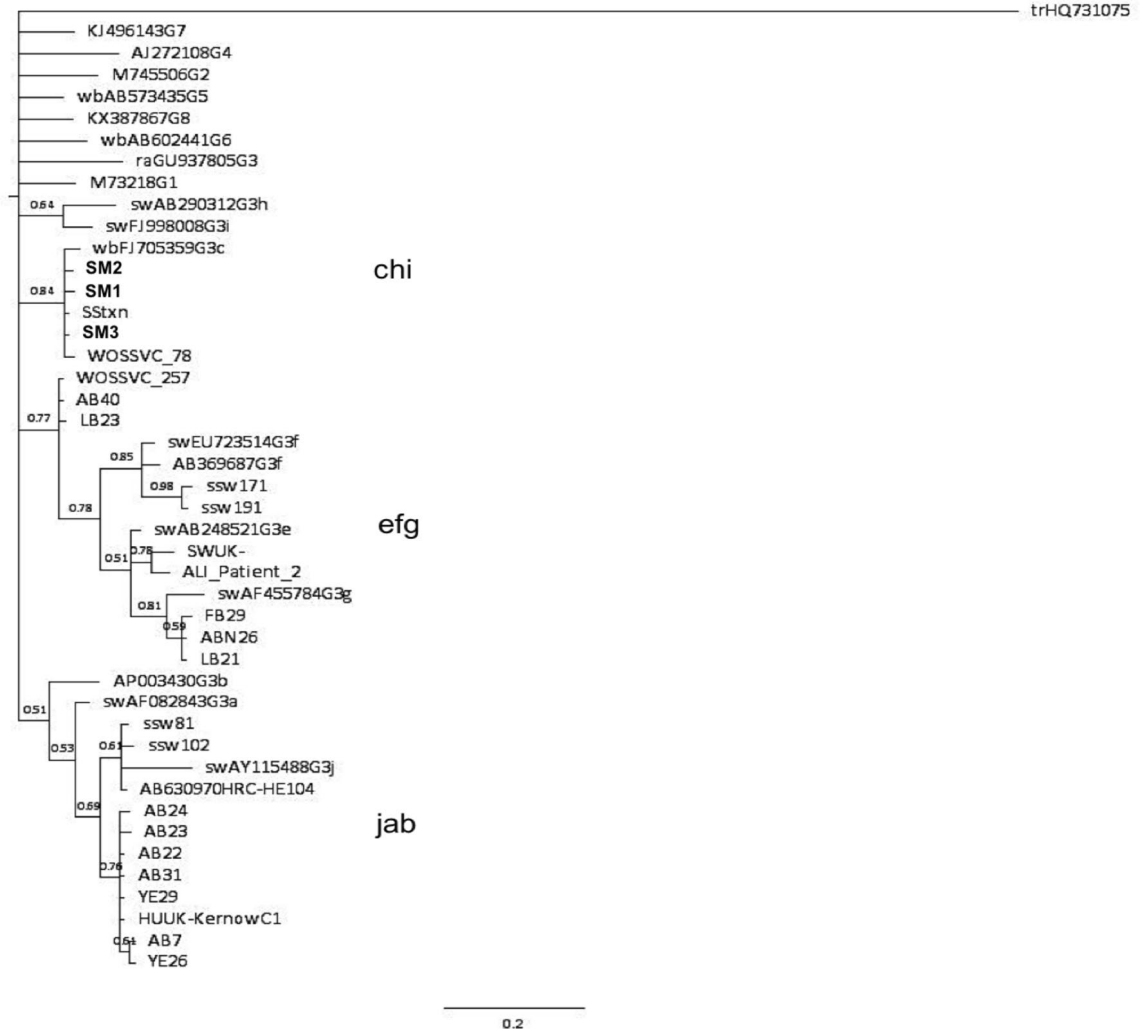
Phylogenetic analysis of 145-nt open reading frame 2 (ORF2) fragments (relative to Burma strain M73218 positions 6390-6534) from Scottish commercial mussels (in bold). Numbers beside nodes indicate posterior probabilities. Reference sequences for all HEV genotypes and subtypes are as follows—genotype 1, M73218; 2, M74506; 3a, AF082843; 3b, AB291955; 3c, FJ705359; 3e, AB248521; 3f, AB369687; 3f, EU723514; 3g, AF455784; 3h, AB290312; 3i, FJ998008; 3j, AY115488; G4, AJ272108; G3 rabbit, GU937805; G5 wild boar, AB5734435; G6 wild boar, AB602441; G7 camel, KJ496143; Cutthroat trout strain Heenan88, HQ389543 (Smith et al. 2016). Additional sequences included in analysis—huUK—human HEV strain C1 Kernow, camel G8, KX8387867. Abbreviations included in tree are as follows—*sw* swine, *ssw* Scottish swine, *wb* wild boar, *ra* rabbit. ORF2 sequences obtained from Scottish wild mussels included in the phylogenetic analysis are named based on their source of origin and are as follows: *AB* Ardrossan beach, *ABN* Aberdeen, *YE* Ythan estuary, *FB* Ferrybridge, *LB* Lunderston Bay (Crossan et al. 2012). ORF2 sequences from Scottish patient sera have also been included in the analysis and are labelled as follows; ALI_patient_2; WOSSVC_78; WOSSVC_257; SStxn (accession number KT159771.1). HEV subtype clusters are indicated by the groupings of letters. Phylogenetic tree analysis was performed using MrBayes version 3.2.1 and the phylogenetic tree generated using Figtree version 1.4.2

Figure 1 shows phylogenetic analysis of ORF2 sequences obtained from mussels along with genotypes 1 to 8 and genotype 3 sequences derived from Scottish wild mussels, swine and patient sera. The mussel sequences are identified in bold and by the codes SM1, SM2 and SM3 (Scottish Mussel 1, 2 and 3).

Phylogenetic analysis of the partial ORF2 sequences shown in Fig. 1 showed that all HEV sequences from the commercial mussels belonged to genotype 3. Moreover, the sequences clustered with the subtype 3c reference sequence from the wild boar strain, wbGER27 (accession number FJ705359). The sequences also clustered with two sequences from human patients, including one patient with a transfusion-transmitted HEV infection (SStxn). Both patients were Scottish and both infections were indigenously acquired. No newly identified shellfish sequences clustered with HEV sequences derived from wild mussels from the 2012 Crossan study (Crossan et al. 2012). Despite many of the wild mussel samples falling within the major clade 1, they did not cluster into the subtype 3c grouping.

## Discussion

Detection of HEV in bivalve mollusks has been reported previously (Li et al. 2007; Song et al. 2010; Donia et al. 2012; Crossan et al. 2012).

The overall HEV prevalence in the Scottish shellfish was 2.9% and was much lower compared to the HEV detection rate in Scottish wild mussels (Crossan et al. 2012). The low prevalence rate reported in this study is very similar to the 2.6% HEV detection rate in shellfish harvested from commercial production sites in Southern Italy (La Rosa et al. 2017). One European study reported that 3% of mussels at retail level from Spain were HEV RNA positive (Diez-Valcarce et al. 2012), whilst another reported 14.8% of mussels harvested from commercial production sites were positive for genotype 3 HEV RNA (Mesquita et al. 2016). Both the former study and the latter study analysed mussels from the Galicia region in Spain but it is unknown whether samples were from the same production site. Another study reported 8.1% of mussels collected in the Tuscany region of Italy were positive for HEV RNA although these were not from approved production sites (Donia et al. 2012). The La Rosa study reported virus titres in mussels being below 10^2^ genome copies/g shellfish (La Rosa et al. 2017). We also report very low virus concentrations in commercial mussels; HEV RNA was quantifiable in only 1 sample with the HEV RNA titre calculated at less than 10^2^ IU/ml. In contrast to the findings reported here and by in the La Rosa study, HEV RNA titres in Scottish wild mussels ranged between 3.73 and 5.2 log_10_ IU/ml (Crossan et al. 2012). The differences observed between the viral titres reported in these studies may reflect that commercially grown shellfish are subject to strict EU hygiene regulations such as those stipulated in (EU) Regulation 853/2004 and (EU) Regulation 854/2004, whilst the wild mussels analysed in the Crossan study were not.

Phylogenetic analysis, although confirming genotype 3, sequences did not cluster with or show any relationship with sequences previously detected in wild mussels or with Scottish pig strains (Crossan et al. 2012, 2014). The wild mussels reported in the 2012 Crossan et al. study were collected from unregulated wild mussel beds in different geographical locations to the shellfish reported in this study. Wild mussels were collected from sites on the northeast coast of Scotland—a region renowned for its pig farming and home to the vast majority of the country’s sows. Furthermore, wild mussels from the west coast were also collected from a unique site; the mussel bed was situated downstream from a pig meat processing plant and in close proximity to a waste outfall pipe, possibly connected to the processing plant (Crossan et al. 2012). Commercial aquaculture sites in Scotland, however, are mainly located on the west coast and the Shetland Isles where pig production is uncommon. Genotype 3 HEV has been reported to fall in to two phylogenetic groups, clade 1 and clade 2 comprising subtypes 3a, 3b, 3c, 3h, 3i and 3j (3abchij) and subtypes 3e, 3f and 3g (3efg), respectively (Smith et al. 2016). The sequences derived from commercial mussels all clustered with the reference subtype 3c sequence, wbGER27, isolated from a wild boar. The commercial shellfish sequences also clustered with genotype 3 HEV sequences obtained from 2 Scottish patients with indigenously acquired genotype 3c HEV infection. It could be posited that the HEV infections of the original blood donor to patient SStxn and WOSSVC_78 may have been acquired via the consumption of contaminated or infected food products such as shellfish or meat products. However, this is speculation and there are currently very limited numbers of studies reporting hepatitis E virus infection following the consumption of contaminated bivalve mollusc shellfish (Koizumi et al. 2004; Li et al. 2007; Said et al. 2009). The three newly obtained sequences described in this study were from shellfish purchased from three separate commercial outlets but within the same time period and it is reasonable to assume that they have come from different aquaculture sites that are widely dispersed around the coast of Scotland, albeit processing is carried out centrally, which could potentially be an alternative source of contamination. It was not possible to draw any conclusions regarding differences in HEV detection during different times of sampling as the study was not designed for this type of analysis (i.e. samples being collected at regular intervals throughout the year).

The presence of HEV in shellfish is likely attributable to contamination of shellfish harvesting waters by human sewage. Many rural dwellings on the west coast of Scotland and on the Shetland Isles do not have adequate sewage treatment systems in place, often resulting in the release of raw or partially treated sewage into the environment. This is surprisingly common according to Scottish Environmental Protection Agency (SEPA) (personal communication), and it is therefore very possible for shellfish harvesting waters to be exposed to enteric viruses, including HEV.

Scotland is home to very large wild deer populations, including red deer and sika deer, and it is therefore possible that animal sewage, including from wild deer, may also be a potential source of contamination as previous studies have found HEV to be circulating amongst deer populations (Tei et al. 2003; Choi et al. 2013; Kukielka et al. 2016). In contrast to wild deer populations in Scotland, the number of wild boar roaming freely in Scotland is very low and widely dispersed, and therefore the risk of contamination of shellfish harvesting sites is significantly lower. It should be noted, however, that studies have suggested that deer are in fact spillover hosts of HEV due to the accidental transmission of HEV from wild boar species, whilst other authors recognise deer as true reservoirs for HEV (Anheyer-Behmenburg et al. 2017; Thiry et al. 2017; Van der Poel 2014). Despite the fact that Scotland produces approximately 3500 tonnes of venison annually and the estimated annual value of Scottish venison sales is £2 million, there are no data regarding the presence of HEV in the Scottish deer population to date or studies examining the risk of acquiring HEV via the consumption of venison meat and processed venison products (Edwards & Kenyon 2013).

The present study is the first to demonstrate the occurrence of genotype 3 HEV in commercially harvested Scottish mussels sold at retail, albeit at extremely low levels (only quantifiable in one sample). Until the dose–response relationship for HEV infection and disease are addressed, the risk to consumers regarding shellfish consumption and the risk of infection is unknown. It is important to note that the detection of viral genomic material by RT-PCR does not distinguish between infectious and non-infectious HEV particles. Until a robust, effective and validated cell-culture method is developed that can determine the infectious capacity of HEV in foodstuffs, it was not possible to determine whether infectious particles were indeed present in the shellfish. Unlike oysters, which are traditionally eaten raw, mussels are less likely to pose a risk of HEV infection to consumers, as they are normally cooked prior consumption. However, the optimum cooking temperatures and the minimum cooking times have yet to be established for complete HEV inactivation in shellfish. Nevertheless, caution should be taken when consuming shellfish, particularly by those deemed to be at higher risk of HEV infection. These findings may encourage further research that will help address the gaps in the knowledge in respect to foodborne transmission of HEV in Scotland and the rest of the United Kingdom.
